# A Prospective Comparative Study of Opioid-Free Anesthesia and Opioid-Based Anesthesia for Postoperative Pain Relief in Patients Undergoing Laparoscopic Surgeries: A Double-Blinded Randomized Controlled Trial

**DOI:** 10.7759/cureus.117045

**Published:** 2026-09-28

**Authors:** Sneha A Johnson, Dilip Kumar Govindan, Easwar Elango, Sony Prakkattumannathu Varghese, Prabakaran P Thamilmani, Jyothi Susan Thomas, Shubhada Deshmukh

**Affiliations:** 1 Department of Anesthesiology, Shri Sathya Sai Medical College and Research Institute, Chennai, IND; 2 Department of Orthopedics, Dr. RK Diabetic Foot and Podiatry Institute, Chennai, IND; 3 Department of Plastic Surgery, Khoula Hospital, Muscat, OMN; 4 Department of General Medicine, Sri Ramaswamy Memorial (SRM) Medical College and Hospital, Trichy, IND; 5 Department of Obstetrics and Gynecology, Badr Al Samaa Hospital, Muscat, OMN; 6 Department of Emergency Medicine, N. K. P. Salve Institute of Medical Sciences and Research Centre and Lata Mangeshkar Hospital, Nagpur, IND

**Keywords:** dexmedetomidine, laparoscopic surgery, multimodal analgesia, opioid-based anaesthesia, opioid-free anaesthesia, postoperative nausea and vomiting, postoperative pain, randomized controlled trial

## Abstract

Background: Opioid-based anesthesia (OBA) is widely used for laparoscopic surgery but may be associated with opioid-related adverse effects, including postoperative nausea and vomiting (PONV), respiratory depression, pruritus, and delayed recovery. This study aimed to evaluate the effectiveness and safety of opioid-free anesthesia (OFA) compared with OBA in patients undergoing elective laparoscopic surgery, with postoperative pain assessed as the primary outcome.

Methods: This double-blind, randomized controlled trial was conducted in the Department of Anesthesiology at Shri Sathya Sai Medical College and Research Institute, Chengalpattu, India. Sixty-two adults undergoing elective laparoscopic surgery were randomly allocated to OFA (n = 31) or OBA (n = 31). Postoperative pain was assessed using the Visual Analog Scale (VAS) at 2, 6, 12, 24, and 48 hours. Secondary outcomes included rescue analgesia, perioperative hemodynamic parameters, PONV, and other postoperative adverse events.

Results: Baseline demographic and clinical characteristics were comparable between the groups. VAS scores were significantly lower with OFA at six hours (3.2 ± 0.8 vs. 4.6 ± 1.2; Holm-adjusted p < 0.001), 12 hours (3.2 ± 0.9 vs. 4.0 ± 0.8; p = 0.004), and 24 hours (3.0 ± 0.6 vs. 3.4 ± 0.4; p = 0.009), whereas scores at 2 and 48 hours were comparable. Rescue analgesia within six hours was required by one patient (3.2%) in the OFA group and six patients (19.4%) in the OBA group (Fisher's exact p = 0.104). Among participants with a recorded rescue-analgesia event, the reported mean time to first rescue analgesia was longer with OFA (8.7 ± 1.9 vs. 7.0 ± 1.7 hours; p < 0.001). Heart rate and systolic blood pressure were comparable between the groups, while diastolic blood pressure and mean arterial pressure were higher with OFA at six hours postoperatively. PONV at six hours and overall complications were less frequent with OFA.

Conclusions: OFA was associated with improved early postoperative analgesia, with lower VAS scores at 6, 12, and 24 hours following elective laparoscopic surgery. Although rescue analgesia within six hours was numerically less frequent with OFA, the difference was not statistically significant. OFA was also associated with lower PONV at six hours and fewer overall complications. Perioperative hemodynamic parameters were broadly comparable, with modest differences in diastolic blood pressure and mean arterial pressure at six hours. Larger multicenter randomized trials are warranted to confirm these findings.

## Introduction

Laparoscopic surgery has become the preferred approach for many abdominal procedures because it is associated with smaller incisions, reduced tissue trauma, shorter hospital stays, faster recovery, and earlier return to daily activities compared with open surgery [1]. Despite these advantages, moderate-to-severe postoperative pain may occur during the first 24 hours after laparoscopic procedures because of port-site trauma, visceral manipulation, pneumoperitoneum, and diaphragmatic irritation, potentially affecting recovery and patient satisfaction [1,2]. Effective perioperative pain management is therefore an essential component of anesthetic care for patients undergoing laparoscopic surgery.

Opioids have traditionally been a cornerstone of balanced general anesthesia because of their potent analgesic effects and ability to attenuate the sympathetic response to surgical stimulation. However, perioperative opioid administration is associated with several adverse effects, including postoperative nausea and vomiting (PONV), sedation, respiratory depression, pruritus, urinary retention, constipation, and delayed recovery [3-5]. Opioid exposure may also contribute to opioid-induced hyperalgesia, tolerance, dependence, and persistent postoperative opioid use [4,5]. These concerns have led to increasing interest in multimodal and opioid-sparing approaches to perioperative analgesia.

Enhanced recovery after surgery pathways emphasize multimodal analgesia to reduce opioid exposure while maintaining adequate pain control and facilitating early postoperative recovery [6]. Opioid-free anesthesia (OFA) is one such approach and avoids the administration of opioids during the intraoperative period by combining nonopioid agents with complementary analgesic mechanisms [6,7]. Depending on the anesthetic protocol, agents such as dexmedetomidine, ketamine, intravenous lignocaine, magnesium sulfate, paracetamol, nonsteroidal anti-inflammatory drugs, and regional or local anesthetic techniques may be incorporated into a multimodal regimen [8]. The rationale for OFA is to provide adequate perioperative nociceptive control while limiting opioid-related adverse effects.

Several clinical studies have evaluated OFA in laparoscopic surgery and have reported potentially favorable postoperative outcomes, including lower pain scores, reduced postoperative opioid or rescue-analgesic requirements, improved recovery, and fewer opioid-related adverse effects [9,10]. Sayed et al. reported favorable postoperative pain and analgesic outcomes with an opioid-free technique, together with differences in perioperative hemodynamic parameters [11]. Other studies have also described earlier recovery, shorter hospital stay, and reduced requirements for antiemetic therapy with opioid-free or opioid-sparing anesthetic approaches [12]. However, findings across studies remain heterogeneous, reflecting differences in anesthetic regimens, surgical procedures, comparator protocols, and outcome definitions. Consequently, the relative benefits and safety of OFA compared with conventional opioid-based anesthesia (OBA) in laparoscopic surgery remain an area of ongoing investigation.

Considering the increasing emphasis on opioid-sparing perioperative care and the need for further prospective randomized evidence in the Indian population, the present double-blinded randomized controlled trial compared OFA with OBA in adults undergoing elective laparoscopic surgery. The primary objective was to compare postoperative pain intensity, assessed using the Visual Analog Scale (VAS) at 2, 6, 12, 24, and 48 hours after surgery. Secondary objectives were to compare the requirement for rescue analgesia, time to first rescue analgesia, perioperative hemodynamic parameters, and postoperative adverse events, including PONV, shivering, pruritus, and bradycardia, between the two groups.

## Materials and methods

Study design and setting

This prospective, double-blinded, randomized controlled trial was conducted at Shri Sathya Sai Medical College and Research Institute from November 2024 to January 2026. The study compared OFA with OBA for postoperative pain control in patients undergoing elective laparoscopic surgery under general anesthesia. The study was approved by the Institutional Human Ethics Committee (IEC No. 984/24) and prospectively registered with the Clinical Trials Registry-India (CTRI/2024/07/071480) before participant enrolment. The study was conducted in accordance with the Declaration of Helsinki, and written informed consent was obtained from all participants. The participant flow through screening, randomization, allocation, follow-up, and final analysis is presented in the Consolidated Standards of Reporting Trials flow diagram (Figure 1).

**Figure 1:**
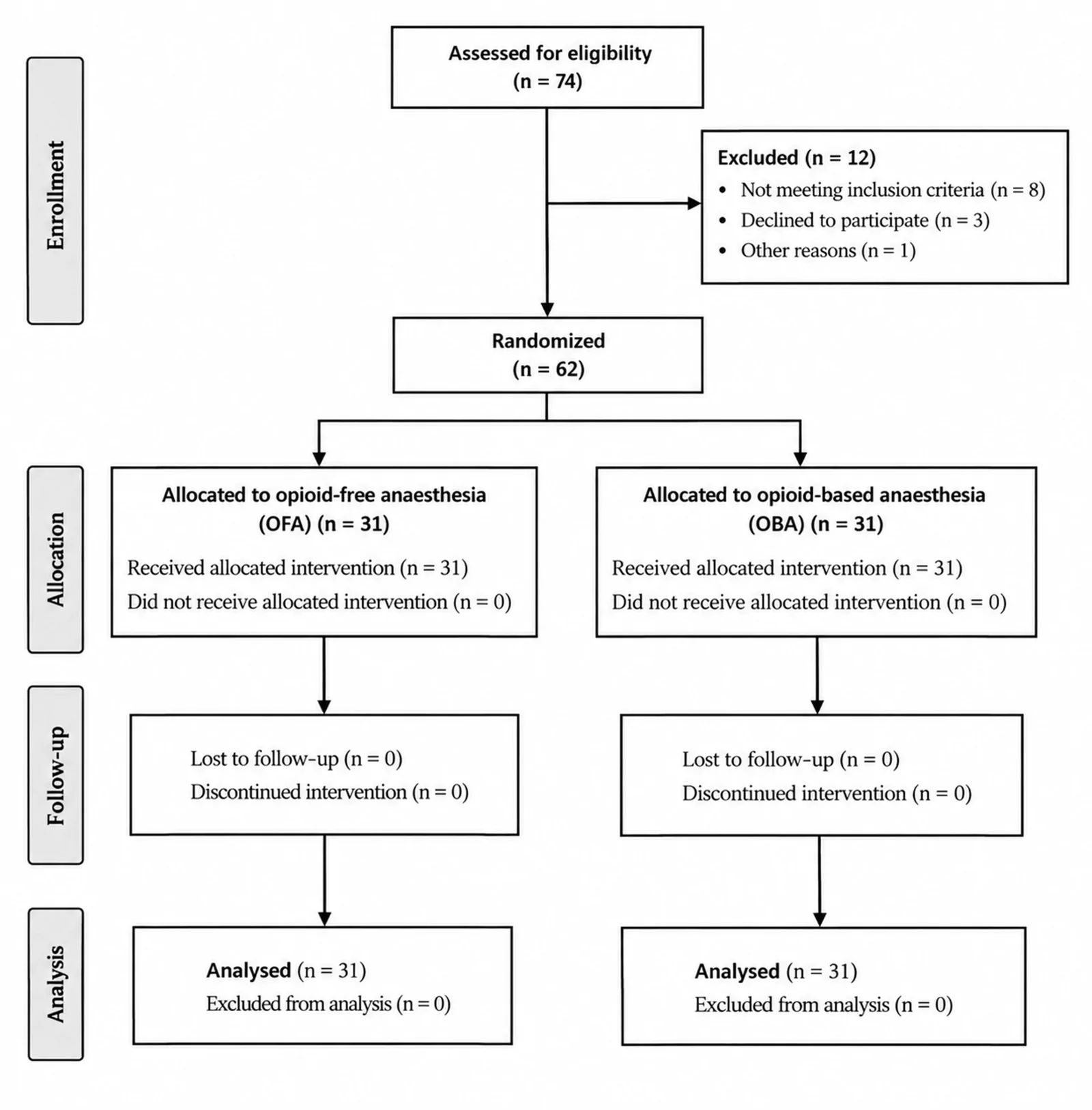
CONSORT flow diagram CONSORT: Consolidated Standards of Reporting Trials; OFA: opioid-free anesthesia; OBA: opioid-based anesthesia

Selection Criteria

Patients aged 18-60 years of either sex, scheduled for elective laparoscopic appendectomy or cholecystectomy under general anesthesia, and classified as American Society of Anesthesiologists (ASA) physical status I-III were eligible. Patients with body mass index (BMI) >35 kg/m², known hypersensitivity to study medications, renal or hepatic failure, hyperthyroidism, current hormone therapy, chronic pain syndrome, psychiatric illness, or cardiovascular disease were excluded. Patients unwilling or unable to provide informed consent were also excluded.

Sample Size

Sample size was calculated using data from a previously published study from Tamil Nadu comparing intraoperative mean arterial pressure (MAP) between OFA and OBA groups [13]. Assuming mean MAP values of 93 and 87 mmHg, a common standard deviation (SD) of 8 mmHg, a two-sided α of 0.05, and 80% power, with an anticipated 10% attrition, the required sample size was estimated at 31 participants per group. Thus, 62 participants were included, with 31 allocated to each group.

Randomization and Blinding

Participants were randomized in a 1:1 ratio to the OFA or OBA group using a computer-generated randomization sequence. Allocation was concealed using sequentially numbered, opaque, sealed envelopes. An independent anesthesiologist prepared the study medications, while the anesthesiologists administering anesthesia, participants, and postoperative outcome assessors remained blinded to group allocation. The allocation envelope was opened immediately before induction by the anesthesiologist responsible for administering the assigned intervention.

Anesthetic Intervention

Patients in the OBA group received intravenous fentanyl 2 µg/kg diluted in 100 mL normal saline 10 minutes before induction, followed by an intraoperative fentanyl infusion of 0.5 µg/kg/hour. Patients in the OFA group received intravenous dexmedetomidine 1 µg/kg, ketamine 0.2 mg/kg, and lignocaine 2 mg/kg diluted in 100 mL normal saline 10 minutes before induction, followed by dexmedetomidine infusion at 0.5 µg/kg/hour.

Both groups received identical general anesthesia apart from the study intervention. Premedication consisted of glycopyrrolate and midazolam. Anesthesia was induced with propofol 2.5 mg/kg, and neuromuscular blockade for tracheal intubation was achieved with succinylcholine 1.5 mg/kg. Anesthesia was maintained with sevoflurane, targeting approximately 1.5 minimum alveolar concentration, with adjustments according to clinical and hemodynamic response. Standard intraoperative monitoring and perioperative care were provided to both groups.

Data collection and outcomes

Baseline demographic and clinical characteristics, including age, sex, BMI, ASA status, and type of laparoscopic procedure, were recorded using a structured case-record proforma. Intraoperative and postoperative heart rate (HR), systolic blood pressure (SBP), diastolic blood pressure (DBP), MAP, peripheral oxygen saturation, respiratory rate, and end-tidal carbon dioxide were recorded at predefined time points.

Postoperative pain was assessed using a 10-cm VAS, ranging from 0 (no pain) to 10 (worst imaginable pain), at 2, 6, 12, 24, and 48 hours. The primary outcome was postoperative pain intensity assessed by VAS. Secondary outcomes included requirement for rescue analgesia, time to first rescue analgesia, perioperative hemodynamic parameters, and postoperative adverse events. PONV, shivering, pruritus, bradycardia, and other clinically relevant complications were recorded during postoperative follow-up. Overall complications were defined as the occurrence of at least one recorded postoperative adverse event.

Statistical analysis

Data were entered into Microsoft Excel (Microsoft Corporation, Redmond, WA) and analyzed using IBM Statistical Package for the Social Sciences (IBM Corp., Armonk, NY). Continuous variables were expressed as mean ± SD, and categorical variables as frequencies and percentages. Baseline continuous variables were compared using the independent-samples t-test, whereas categorical variables were analyzed using the chi-square test or Fisher's exact test, as appropriate.

Repeated VAS and hemodynamic measurements were analyzed using a repeated-measures model incorporating treatment group, time, and group-by-time interaction. Post hoc between-group comparisons at individual time points were adjusted for multiple comparisons using the Holm method. Fisher's exact test was used for categorical outcomes with small expected cell counts. Time to first rescue analgesia was compared between the groups based on the observed time to first rescue request. All tests were two-sided, and p < 0.05 was considered statistically significant. Where appropriate, treatment effects were reported with 95% confidence intervals (CIs).

## Results

A total of 62 participants were randomized, with 31 participants allocated to the OFA group and 31 to the OBA group. Baseline demographic and clinical characteristics are presented in Table 1. There were no statistically significant between-group differences in age, sex, BMI, ASA physical status, type of laparoscopic surgery, or duration of surgery. The mean age was 35.1 ± 11.1 years in the OFA group and 37.2 ± 10.5 years in the OBA group (p = 0.447). Male participants comprised 17 (54.8%) in the OFA group and 20 (64.5%) in the OBA group (p = 0.437). Mean BMI was 24.1 ± 2.5 kg/m² in the OFA group and 24.5 ± 2.0 kg/m² in the OBA group (p = 0.489). The distribution of ASA physical status was comparable between the groups (p = 0.677). Laparoscopic appendicectomy was performed in 12 (38.7%) participants in the OFA group and 17 (54.8%) in the OBA group, whereas laparoscopic cholecystectomy was performed in 19 (61.3%) and 14 (45.2%), respectively (p = 0.203). The mean duration of surgery was 83.1 ± 15.3 minutes in the OFA group and 84.2 ± 9.8 minutes in the OBA group (p = 0.737).

**Table 1:** Baseline demographic and clinical characteristics of the study participants Values are presented as mean ± SD or n (%). Between-group comparisons were performed using the independent-samples t-test for continuous variables and the chi-square test for categorical variables BMI: body mass index; ASA: American Society of Anesthesiologists; OFA: opioid-free anesthesia; OBA: opioid-based anesthesia; SD: standard deviation

Characteristic	OFA (n = 31)	OBA (n = 31)	p value
Age, years	35.1 ± 11.1	37.2 ± 10.5	0.447
Male	17 (54.8%)	20 (64.5%)	0.437
Female	14 (45.2%)	11 (35.5%)	
BMI, kg/m²	24.1 ± 2.5	24.5 ± 2.0	0.489
ASA I	20 (64.5%)	18 (58.1%)	0.677
ASA II	8 (25.8%)	11 (35.5%)	
ASA III	3 (9.7%)	2 (6.5%)	
Laparoscopic appendicectomy	12 (38.7%)	17 (54.8%)	0.203
Laparoscopic cholecystectomy	19 (61.3%)	14 (45.2%)	
Duration of surgery, minutes	83.1 ± 15.3	84.2 ± 9.8	0.737

Perioperative HR, SBP, DBP, and MAP are presented in Table 2. HR and SBP were comparable between the two groups across the assessed perioperative time points.

**Table 2:** Perioperative hemodynamic parameters Values are presented as mean ± SD. Between-group comparisons were performed using the independent-samples t-test HR: heart rate; SBP: systolic blood pressure; DBP: diastolic blood pressure; MAP: mean arterial pressure; OFA: opioid-free anesthesia; OBA: opioid-based anesthesia; SD: standard deviation

Time point	Parameter	OFA (n = 31)	OBA (n = 31)	p value
Baseline	HR, beats/minute	82.1 ± 2.2	83.1 ± 2.3	0.085
	SBP, mmHg	110.8 ± 5.4	111.9 ± 2.8	0.318
	DBP, mmHg	71.3 ± 4.3	71.8 ± 2.5	0.578
	MAP, mmHg	84.5 ± 3.4	85.2 ± 1.9	0.320
After intubation	HR, beats/minute	83.5 ± 3.5	84.2 ± 5.4	0.547
	SBP, mmHg	112.8 ± 4.9	110.9 ± 8.7	0.294
	DBP, mmHg	70.5 ± 5.8	71.2 ± 1.1	0.512
	MAP, mmHg	84.6 ± 4.2	84.4 ± 3.0	0.858
Before pneumoperitoneum	HR, beats/minute	83.2 ± 5.5	82.4 ± 5.5	0.569
	SBP, mmHg	110.5 ± 5.8	111.2 ± 2.9	0.550
	DBP, mmHg	71.5 ± 3.9	70.9 ± 2.9	0.494
	MAP, mmHg	84.5 ± 3.2	84.3 ± 2.2	0.812
10 minutes after pneumoperitoneum	HR, beats/minute	84.2 ± 5.3	84.7 ± 4.8	0.698
	SBP, mmHg	108.4 ± 10.4	106.5 ± 6.7	0.396
	DBP, mmHg	72.8 ± 4.7	71.8 ± 3.5	0.346
	MAP, mmHg	84.7 ± 4.7	83.4 ± 3.2	0.208
After CO₂ deflation	HR, beats/minute	83.5 ± 5.9	83.6 ± 5.9	0.947
	SBP, mmHg	110.6 ± 5.8	110.5 ± 2.2	0.929
	DBP, mmHg	73.2 ± 4.5	71.5 ± 2.7	0.076
	MAP, mmHg	85.7 ± 3.6	84.5 ± 1.9	0.115
After extubation	HR, beats/minute	84.1 ± 7.0	82.8 ± 5.1	0.407
	SBP, mmHg	112.3 ± 4.8	110.9 ± 7.5	0.385
	DBP, mmHg	72.4 ± 3.7	71.9 ± 2.5	0.535
	MAP, mmHg	85.7 ± 2.9	84.9 ± 3.0	0.294
Postoperative 6 hours	HR, beats/minute	82.5 ± 5.5	82.2 ± 5.8	0.835
	SBP, mmHg	111.5 ± 5.3	109.7 ± 4.1	0.140
	DBP, mmHg	73.8 ± 2.9	72.0 ± 2.4	0.010
	MAP, mmHg	86.4 ± 2.6	84.6 ± 2.1	0.004
Postoperative 24 hours	HR, beats/minute	80.8 ± 7.5	80.6 ± 5.1	0.903
	SBP, mmHg	111.1 ± 5.8	109.4 ± 2.1	0.130
	DBP, mmHg	72.2 ± 3.4	72.1 ± 2.8	0.900
	MAP, mmHg	85.2 ± 3.0	84.5 ± 2.0	0.329

A statistically significant between-group difference in DBP was observed at six hours postoperatively, with a higher mean DBP in the OFA group than in the OBA group (73.8 ± 2.9 vs. 72.0 ± 2.4 mmHg, p = 0.010). No statistically significant differences in DBP were observed at the other assessed time points. MAP was higher in the OFA group at six hours postoperatively (86.4 ± 2.6 vs. 84.6 ± 2.1 mmHg, p = 0.004). No statistically significant between-group differences in MAP were observed at the remaining assessed time points.

Postoperative pain was assessed using the VAS at 2, 6, 12, 24, and 48 hours. The between-group comparisons are presented in Table 3. At two hours, VAS scores were comparable between the OFA and OBA groups (3.0 ± 1.4 vs. 3.2 ± 2.9; mean difference, -0.20; 95% CI, -1.37 to 0.97; Holm-adjusted p = 0.731). At six hours, VAS scores were significantly lower in the OFA group (3.2 ± 0.8 vs. 4.6 ± 1.2; mean difference, -1.40; 95% CI, -1.92 to -0.88; Holm-adjusted p < 0.001). Similarly, significantly lower VAS scores were observed in the OFA group at 12 hours (3.2 ± 0.9 vs. 4.0 ± 0.8; mean difference, -0.80; 95% CI, -1.23 to -0.37; Holm-adjusted p = 0.004), and 24 hours (3.0 ± 0.6 vs. 3.4 ± 0.4; mean difference, -0.40; 95% CI, -0.66 to -0.14; Holm-adjusted p = 0.009). At 48 hours, the difference between groups was not statistically significant (2.7 ± 0.5 vs. 2.9 ± 0.5; mean difference, -0.20; 95% CI, -0.45 to 0.05; Holm-adjusted p = 0.121).

**Table 3:** Postoperative VAS pain scores Values are presented as mean ± SD. Between-group comparisons were performed using the independent-samples t-test, with Holm adjustment for multiple comparisons across the five postoperative time points. Mean differences are presented as OFA minus OBA VAS: visual analog scale; CI: confidence interval; OFA: opioid-free anesthesia; OBA: opioid-based anesthesia; SD: standard deviation

Time point	OFA (n = 31)	OBA (n = 31)	Mean difference (OFA-OBA)	95% CI	Holm-adjusted p value
2 hours	3.0 ± 1.4	3.2 ± 2.9	-0.20	-1.37 to 0.97	0.731
6 hours	3.2 ± 0.8	4.6 ± 1.2	-1.40	-1.92 to -0.88	<0.001
12 hours	3.2 ± 0.9	4.0 ± 0.8	-0.80	-1.23 to -0.37	0.004
24 hours	3.0 ± 0.6	3.4 ± 0.4	-0.40	-0.66 to -0.14	0.009
48 hours	2.7 ± 0.5	2.9 ± 0.5	-0.20	-0.45 to 0.05	0.121

Rescue analgesia outcomes are presented in Table 4. Within the first six postoperative hours, rescue analgesia was required by one participant (3.2%) in the OFA group and six participants (19.4%) in the OBA group. The proportion requiring rescue analgesia was numerically lower in the OFA group; however, the difference was not statistically significant on Fisher's exact test (p = 0.104). Among participants with a recorded rescue-analgesia event, the reported mean time to first rescue analgesia was 8.7 ± 1.9 hours in the OFA group compared with 7.0 ± 1.7 hours in the OBA group (p < 0.001). This comparison represents the observed rescue times among participants with recorded rescue events.

**Table 4:** Rescue analgesia outcomes Values are presented as n (%) or mean ± SD. The between-group comparison for rescue analgesia requirement was performed using Fisher's exact test, and the comparison of mean time to first rescue analgesia was performed using the independent-samples t-test OFA: opioid-free anesthesia; OBA: opioid-based anesthesia; SD: standard deviation

Outcome	OFA (n = 31)	OBA (n = 31)	p value
Rescue analgesia within 6 hours	1 (3.2%)	6 (19.4%)	0.104
Mean time to first rescue analgesia, hours	8.7 ± 1.9	7.0 ± 1.7	<0.001

Postoperative adverse events, including PONV, shivering, pruritus, and bradycardia, are presented in Table 5. PONV at 0 hours occurred in two participants (6.5%) in the OFA group and six participants (19.4%) in the OBA group (p = 0.255). At six hours, PONV occurred in zero participants in the OFA group compared with six participants (19.4%) in the OBA group, representing a statistically significant between-group difference (p = 0.024). At 24 hours, PONV occurred in zero participants in the OFA group and four participants (12.9%) in the OBA group (p = 0.113).

**Table 5:** Postoperative adverse events Values are presented as n (%). Between-group comparisons were performed using Fisher's exact test PONV: postoperative nausea and vomiting; OFA: opioid-free anesthesia; OBA: opioid-based anesthesia

Adverse event	OFA (n = 31)	OBA (n = 31)	Fisher's exact p value
PONV at 0 hours	2 (6.5%)	6 (19.4%)	0.255
PONV at 6 hours	0	6 (19.4%)	0.024
PONV at 24 hours	0	4 (12.9%)	0.113
Shivering at 6 hours	0	4 (12.9%)	0.113
Pruritus	0	4 (12.9%)	0.113
Bradycardia	1 (3.2%)	4 (12.9%)	0.354
Overall complications	1 (3.2%)	8 (25.8%)	0.026

Shivering at six hours was observed in zero participants in the OFA group and four participants (12.9%) in the OBA group (p = 0.113). Pruritus occurred in zero participants in the OFA group and four participants (12.9%) in the OBA group (p = 0.113). Bradycardia occurred in one participant (3.2%) in the OFA group and four participants (12.9%) in the OBA group (p = 0.354).

We defined the composite outcome of overall complications as the occurrence of at least one recorded postoperative adverse event included in the study's complication assessment. Each participant was counted once in the composite outcome, irrespective of the number of individual adverse events experienced. Overall complications occurred in one participant (3.2%) in the OFA group and eight participants (25.8%) in the OBA group (Fisher's exact p = 0.026).

## Discussion

The present randomized controlled study evaluated an opioid-free anesthetic technique incorporating dexmedetomidine, ketamine, and lignocaine in patients undergoing laparoscopic surgery and compared it with conventional OBA. The principal finding was that OFA was associated with lower postoperative pain scores at 6, 12, and 24 hours, while pain scores at 2 and 48 hours were comparable between the groups. The proportion of patients requiring rescue analgesia within the first six hours was numerically lower with OFA but did not reach statistical significance on Fisher's exact test. Among patients with a recorded rescue-analgesia event, the reported time to first rescue analgesia was longer in the OFA group. OFA was also associated with fewer PONV events at six hours and a lower overall complication rate. Hemodynamic parameters were generally comparable, although DBP and MAP were significantly higher in the OFA group at six hours postoperatively. Thus, the findings support improved early postoperative pain control with OFA, while the hemodynamic findings should be interpreted as showing broadly comparable profiles with some between-group differences rather than complete hemodynamic equivalence.

The similarity in baseline characteristics supports the comparability of the two treatment groups. There were no significant differences in age, sex distribution, BMI, ASA physical status, type of laparoscopic procedure, or duration of surgery. Similar baseline comparability has been reported in previous randomized comparisons of OFA and OBA, including studies by Sayed et al., Ragupathy et al., and Bora et al. [11,13,14]. The balanced distribution of these characteristics reduces the likelihood that baseline demographic or procedural differences substantially influenced the observed postoperative outcomes.

Maintenance of perioperative hemodynamic stability is an important consideration when evaluating opioid-free anesthetic techniques. In the present study, HR and SBP did not differ significantly between groups at any assessed time point. DBP was significantly higher in the OFA group at six hours postoperatively, and MAP was also higher at this time point. At the remaining assessment points, no significant between-group differences were observed for DBP or MAP. These differences were relatively small in absolute magnitude and did not indicate clinically important hemodynamic instability. Previous studies by Hao et al. and Regis and Mariappan have similarly reported generally acceptable hemodynamic profiles with opioid-free techniques incorporating multimodal agents in laparoscopic surgery [15,16]. However, given the significant differences observed at six hours in the present study, the findings should be interpreted as demonstrating overall hemodynamic stability with some between-group variation rather than evidence of complete hemodynamic equivalence.

A major finding of the present study was the improvement in early postoperative pain control with OFA. Patients receiving OFA had lower VAS scores at 6, 12, and 24 hours than those receiving OBA, with the largest between-group difference observed at six hours. The differences were maintained after adjustment for multiple comparisons, supporting the consistency of the early postoperative analgesic benefit. In contrast, no statistically significant differences were observed at 2 or 48 hours, suggesting that the benefit of the OFA protocol was most apparent during the early postoperative period rather than throughout the entire 48-hour follow-up period.

The reported mean time to first rescue analgesia was also longer in the OFA group among participants with recorded rescue events. However, the proportion requiring rescue analgesia within the first six hours was not significantly different between groups when analyzed using Fisher's exact test. Therefore, the present findings support a difference in postoperative pain scores and suggest a longer observed interval before rescue analgesia among those who required it, but they do not provide definitive evidence of a reduction in the overall proportion of patients requiring rescue analgesia. These findings are broadly consistent with previous reports. Berlier et al. reported reduced morphine requirements following OFA, while Yu et al. observed lower analgesic consumption and improved recovery following laparoscopic procedures [17,18]. Ragupathy et al. similarly reported lower postoperative pain scores and prolonged analgesic duration with OFA [13]. In addition, the systematic review and meta-analysis by Cheng et al. reported improved early postoperative pain control and reduced analgesic requirements with OFA in laparoscopic surgery, although heterogeneity among the included studies should be considered when comparing their findings with the present study [19].

Another relevant finding was the lower frequency of postoperative adverse events in the OFA group. PONV was significantly less frequent in the OFA group at six hours, although differences at 0 and 24 hours did not reach statistical significance. Shivering and pruritus were also numerically less frequent with OFA, but these individual comparisons were not statistically significant. Bradycardia did not differ significantly between the groups. The composite overall complication rate was significantly lower in the OFA group. These findings are clinically relevant because avoidance of perioperative opioids may reduce exposure to adverse effects commonly associated with opioid-based analgesia, particularly nausea, vomiting, pruritus, and sedation. Previous studies by Vishnuraj et al. and Accurso et al. have also reported favorable postoperative recovery profiles and reductions in PONV with opioid-free or opioid-minimizing anesthetic approaches [20,21]. Nevertheless, the individual adverse-event findings in the present study should be interpreted cautiously because the sample size was relatively small and several events occurred infrequently.

The findings of this study therefore add to the growing evidence supporting multimodal OFA as an approach to perioperative analgesia in laparoscopic surgery. The combination of agents with complementary pharmacological mechanisms may provide effective analgesia while reducing reliance on intraoperative opioids. The observed reduction in early postoperative pain and lower frequency of selected adverse events are consistent with the principles of enhanced recovery pathways, in which multimodal and opioid-sparing strategies are used to facilitate postoperative recovery. However, the present study did not demonstrate a statistically significant reduction in the proportion of patients requiring rescue analgesia within six hours; therefore, the findings should not be interpreted as definitive evidence that OFA eliminates the need for postoperative rescue analgesia.

Strengths and limitations

The strengths of this study include its prospective double-blinded randomized controlled design, prospective registration with the Clinical Trials Registry-India, standardized anesthetic protocols, and assessment of multiple clinically relevant postoperative outcomes. Randomization resulted in comparable baseline characteristics between the two groups, strengthening the validity of the comparative analysis. The assessment of postoperative pain at multiple clinically relevant time points also allowed the temporal pattern of analgesic benefit to be evaluated.

The study has several limitations. It was conducted at a single tertiary care center and included a relatively small sample, which may limit the generalizability of the findings. Patients with BMI >35 kg/m² and significant comorbidities were excluded; therefore, the findings may not be directly applicable to patients at higher perioperative risk. The postoperative follow-up was limited to 48 hours and did not permit assessment of longer term outcomes such as chronic postoperative pain, prolonged opioid use, or long-term quality of recovery. In addition, the sample-size calculation was based on an anticipated difference in MAP, whereas postoperative VAS was the prespecified primary outcome. The available analysis also relied on pointwise comparisons of postoperative VAS scores rather than a patient-level repeated-measures model, limiting assessment of the overall group-by-time trajectory. Finally, the relatively low frequency of several adverse events resulted in sparse categorical data and limited the precision of between-group comparisons. Larger, multicenter randomized studies with longer follow-up and appropriately powered longitudinal analyses are warranted to confirm the analgesic and safety benefits of OFA in laparoscopic surgery.

## Conclusions

OFA was associated with significantly better early postoperative pain control than conventional OBA, with lower VAS scores at 6, 12, and 24 hours after laparoscopic surgery. Although fewer patients required rescue analgesia within the first six hours in the OFA group, this difference was not statistically significant. Among participants with recorded rescue-analgesia events, the reported time to first rescue analgesia was longer with OFA. OFA was also associated with a lower incidence of PONV at six hours and fewer overall complications. Perioperative HR and SBP remained comparable between groups, while the higher DBP and MAP observed in the OFA group at six hours were small in absolute magnitude. Overall, these findings support OFA as a potentially effective multimodal approach for improving early postoperative analgesia while reducing exposure to opioid-related adverse effects. Larger multicenter randomized studies with longer follow-up are warranted to confirm these findings and determine their applicability across broader surgical populations.
